# Obstetric-related lower back pain: the effect of number of pregnancy on development of chronic lower back pain, worsening of lumbar disc degeneration and alteration of lumbar sagittal balance

**DOI:** 10.1186/s13018-024-04647-6

**Published:** 2024-03-07

**Authors:** Erdal Güngör, Zeynep Karakuzu Güngör

**Affiliations:** 1 Batman Training and Research Hospital, Department of Orthopaedic and Traumatology, Batman, Turkey; 2 Batman Training and Research Hospital, Department of Physical Medicine and Rehabilitation, Batman, Turkey

**Keywords:** Grand multipara, Low back pain, Parity, Sagittal balance parameters

## Abstract

**Objective:**

This study aims to determine whether the number of pregnancies contributes to the development of chronic lower back pain, worsening the lumbar disc degeneration and altering the normal lumbar sagittal balance.

**Material Method:**

There are 134 ladies participated in this study. They are divided into two groups based on their number of pregnancies (parity). All patients with chronic back pain were assessed using a visual analog scale for pain and the Oswestry Disability Index for their functional status assessment. Degenerative signs in lumbar MRI, which are Modic changes and the presence of Schmorl's node, were evaluated. Besides that, the sagittal balance of the lumbar spine was also measured via an erect lumbar plain radiograph.

**Results:**

Patients with parities < 5 were included in Group 1, and those with parities ≥ 5 in Group 2. The mean visual analog scale score of Group 2 was significantly higher than that of Group 1 (8.42 ± 1.34 vs.6.50 ± 1.61). The mean Oswestry Disability Index score in Group 2 was significantly higher than that of Group 1 (29.87 ± 6.75 vs.18.41 ± 7.97). This relationship between the groups in terms of Modic change was statistically significant. The relationship between the groups regarding the presence of Schmorl’s nodes was also statistically significant. The difference between the groups in terms of sagittal balance parameters was not statistically significant.

**Conclusion:**

Chronic lower back pain is significantly worse and associated with more disability in patients with more than five previous pregnancies. MRI degenerative changes are also significantly higher in these grand multipara groups.

## Introduction

Back pain is a major problem in the general population [1], and it is thought to be even more common in pregnant women who have given birth. It has been hypothesized that this type of pregnancy-related low back pain (LBP) may be related to changes in lumbar posture, perhaps accompanied by stretching abdominal muscles or specific hormonal effects of pregnancy [2]. Recent evidence suggests that sex hormones affect the severity of disc degeneration and may cause vertebral endplate signal changes (VESC) [3]. Estrogen and progesterone hormone receptors are expressed in the end plate cartilage tissue [4], suggesting that cartilage may respond to sex hormones. Therefore, changes in the rate of estrogen release during pregnancy can significantly affect the risk of developing VESC and degenerative disc disease. Estrogen deficiency increases the risk of disc degeneration during the postmenopausal period [3]. Therefore, many studies have shown that postmenopausal estrogen deficiency negatively affects the quality of the vertebral end plates and causes degenerative disc disease.

A few studies have shown a positive relationship between the number of full-term pregnancies or the total number of children a woman experiences and the prevalence of subsequent LBP [5–8]. However, a lower prevalence was also observed among women with multiple children [9]. In contrast, a large population-based, a cross-sectional study found an increased prevalence of LBP among women who had previously been pregnant. However, no trend was seen based on the number of pregnancies [10]. Based on that fact, we are trying to observe the relation between the severity of lower back pain, degenerative changes, and lumbar sagittal balance with the number of pregnancies our patients experienced. The complexity of managing LBP, especially during and after pregnancy, necessitates a multidisciplinary approach, as underscored by recent studies. For instance, Migliorini et al. [11] advocate for a nuanced pharmacological strategy, emphasizing non-pharmacological methods as a primary therapy. Additionally, Baroncini et al. [12] reveal the potential of acupuncture as a significant non-pharmacological intervention for chronic LBP. Furthermore, interventions targeting facet joint osteoarthritis and the utilization of nonopioid pharmacological management, as discussed in subsequent reviews by Baroncini et al. [13], offer promising avenues for comprehensive LBP management, reflecting the need for individualized, multifaceted treatment plans [14]."

## Methodology

Study design: Cross-sectional Observational study.

### Patients and methods

There are 134 ladies participated in this study. All of them has been diagnosed to have chronic lower back pain for various reason. They experienced the pain for at least three months and were investigated radiologically. All of them had experienced various numbers of pregnancies.

They are divided then into two groups based on their number of pregnancies (parity). The first group is called a non-grand multipara for the lady with less than five pregnancies. The second group is a grand multipara for the lady with five or more pregnancies. The subjects are taken between the year 2018 to 2021. The age of the participant in this study is between 18 to 75 years old. The patient who is experienced stillbirth is also counted in the number of pregnancies.

A reasonable definition of "grand multiparity" is a patient who has had ≥ 5 births (live or stillborn) at ≥ 20 weeks of gestation, with "great grand multiparity" defined as ≥ 10 births (live or stillborn) ≥ 20 weeks of gestation [15].

Patients with lumbal trauma, postmenopausal osteoporosis, metabolic diseases, infections, chronic inflammatory conditions, spinal tumors, marked/severe spinal deformities, rheumatoid arthritis, vertebral fractures, lumbar ruptured or herniated disc, or a history of lumbar or cervical spine surgery, and those with surgery traumas, history of depression, low back pain before pregnancy, patients without direct X-ray and MR imaging were excluded from the study. Only patients with nonspecific low back pain were evaluated. Patients with hip and upper lumbar pain were not included in the study. In light of studies conducted, we have observed that the most common causes of lower back pain in women are vertebral deformity and osteoporosis. As specified in inclusion and exclusion criteria of our own study, we focused on patients without vertebral deformity and osteoporosis, aiming to explore the extent to which pregnancy can affect low back pain in these patients.

The lower back pain's severity was assessed using a visual analog score (VAS). In contrast, the functional disability due to pain is evaluated by using Oswestry Disability Index (ODI).

Radiological images and data were reviewed retrospectively. The presence of Modic changes and Schmorl’s nodes were evaluated on MRI, and sagittal balance parameters and spondylolisthesis were measured on lumbar plain radiograph by both authors at first.

The usual demographic parameters, including age, BMI, and disease duration, have also been documented in the result.

The study protocol was conducted according to the Declaration of Helsinki and approved by the local ethical committee of our hospital (Decision no: 281, Date: October 08, 2021).

#### Visual analog scale (VAS)

A visual analog scale (VAS) was used to evaluate the pain severity (0 = no pain, 10 = most severe pain) on a 10-cm-line. The patients were asked to mark their pain level while resting and during activity (functional pain) [16].

#### Oswestry disability index

The Oswestry Disability Index (ODI, also known as the Oswestry Low Back Pain Disability Questionnaire) is an essential tool to measure a patient's permanent functional disability. The test is the gold standard of low-back functional outcome tools [17]. VAS and ODI were evaluated at the first examination.

#### Sagittal balance parameters that matter to analyze the spinopelvic complex

**Fig. 1:**
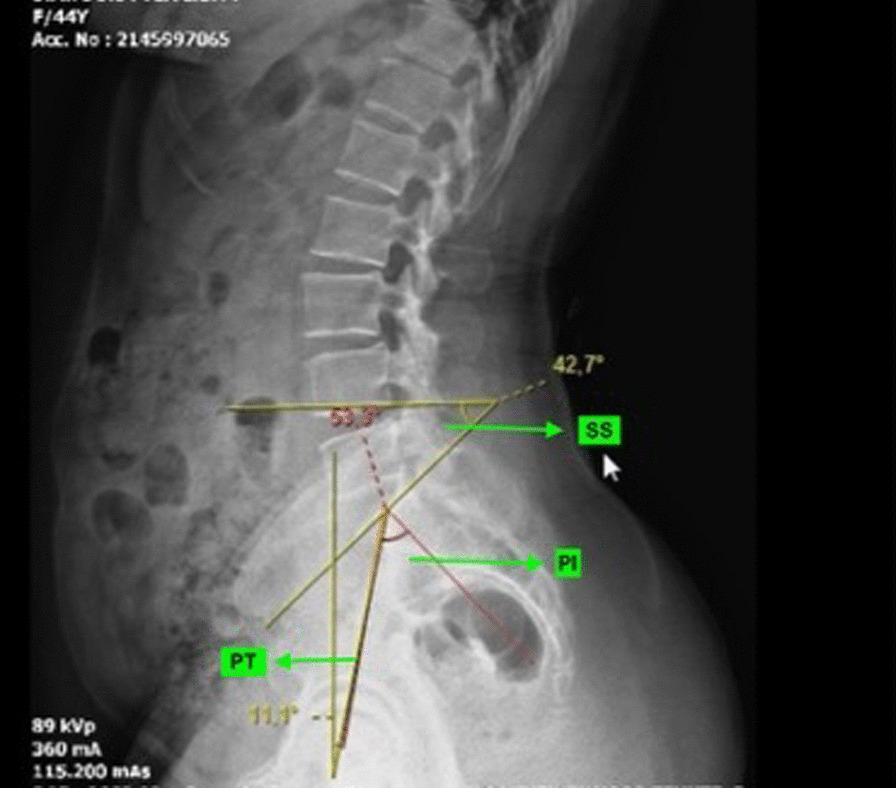
PI; Pelvic incidence PT; pelvic tilt SS; sacral slope

Pelvic incidence is the first pelvic parameter to consider in evaluating the sagittal balance. As described by Legaye et al. and Duval-Beaupère et al. pelvic incidence corresponds to the angle between the line perpendicular to the upper S1 level passing through its center and the line connecting this point to the axis of the femoral heads [18, 19] (Fig. 1). It is directly related to the value of the lumbar lordosis and ranges from 34° to 84°, with an average of 52°[18, 20, 21]

The sacral slope is the angle between a line tangent to the upper-end plate of S1 and the horizontal plane. A vertical pelvis implies a low sacral slope, while a horizontal pelvis would have a high slope. It varies between 20° and 65°, with an average of 40°[20] The pelvic tilt is defined as the angle between a vertical reference line and the line connecting the center of the sacral end plate to the axis of the femoral heads. Those two angles are positional and related to the orientation of the pelvis. It varies between 5° and 30°, with an average of 12°°[18, 20, 21] The pelvic incidence is equal to the arithmetic sum of the sacral slope and the pelvic tilt (PI = PT + SS). A patient with a high pelvic incidence angle has a greater potential for pelvic retroversion. This is an essential piece of information when analyzing compensatory mechanisms.

#### Lumbar parameters

**Fig. 2:**
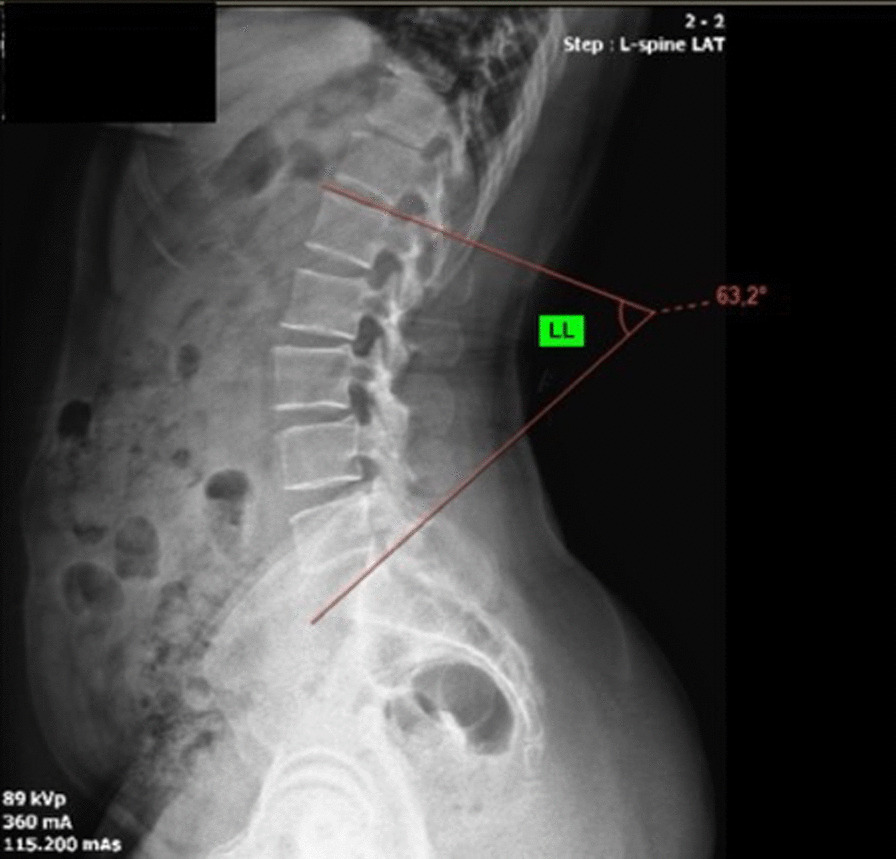
Lumbar parameters. LL; Lumbar lordosis

According to Roussouly [22] et al., lumbar lordosis (LL) is measured between the point of inflection from lumbar lordosis to thoracic kyphosis and the upper-end plate of S1. This point is geometrically calculated when lumbar lordosis turns to thoracic kyphosis It normally ranges between 30° and 79° [23–25] (Fig. 2).

#### Spondylolisthesis

Spondylolisthesis was developed to address this need and is graded on a I to V scale according to the severity of the slip, as determined with plain radiographs by the Meyerding classification system. The Meyerding classification grade is determined by measuring the degree of slip using standing, neutral lateral radiographs of the lumbar spine [26]. The classification system divides slip into five grades: 0% to 25% is Grade I, 25% to 50% is Grade II, 50% to 75% is Grade III, 75% to 100% is Grade IV, and greater than 100% is Grade V [27].

#### Statistics

Statistical analyses of the study were performed using the SPSS v.25.0 software for Windows (IBM Corp., Armonk, NY, USA). The normality assumption was tested with the Kolmogorov–Smirnov and Shapiro–Wilk tests. Explanatory statistics of the variables are given as mean ± standard deviation and n (%). The independent t-test, ANOVA, the Chi-square test, and the Fisher-Freeman-Halton test were used for univariate analyses, depending on the variable type and the assumptions' availability. When a significant difference was detected between the groups as a result of ANOVA, Duncan's multiple range test was used to determine the group that caused the difference. All statistical comparisons were tested in two ways, and cases with a p-value below 0.05 were interpreted as statistically significant.

## Results

The descriptive statistics and group comparison results of the participants are summarized in Table 1. The mean age of the patients was 48.49 ± 9.46 years. Patients who had experienced four or fewer deliveries were included in Group 1, and those who had experienced five or more deliveries in Group 2. 68 patients were included in group 1 and 66 in group 2. The mean age of the patients in Group 1 was 47.94 ± 8.07 years, whereas the mean age in Group 2 was 48.15 ± 6.42 years, exhibiting a not significant difference (*p* = 0.552). The mean BMI of the patients was 28.71 ± 3.54 in Group 1 and 30.19 ± 3.82 in Group 2. Again, the difference between the groups was not statistically significant (*p* = 0.440). The mean VAS score in Group 2 was significantly higher than the mean VAS score in Group 1 (8.42 ± 1.34 vs. 6.50 ± 1.61), exhibiting a more significant difference (*p* < 0.01). The groups also showed a more significant difference in disease duration (*p* < 0.01). As for the ODI, the mean index score in Group 2 was higher than that in Group 1 (29.87 ± 6.75 vs. 18.41 ± 7.97), and this difference was statistically more significant (*p* < 0.01). There were no Modic changes in 25% of the patients in Group 1. The remaining 55.4% had Type 1, and 20.6% had Type 2 Modic changes.Type 2 Modic changes were the most prevalent type in Group 2 (48.5%), closely followed by Type 1 change (45.5%), and only 6.1% of the patients did not have any modic changes. The relationship between the groups in Modic change was statistically significant (*p* < 0.05). Most patients in Group 1 (67.6%) did not have Schmorl's nodes, whereas the situation was the opposite for Group 2 (60.6%). This shows that Schmorl's nodes were also observed in patients with high pregnancies (Group 2). The relationship between the groups in terms of Schmorl's nodes was also statistically significant (*p* < 0.05). The difference between the groups in other variables was not statistically significant.

**Table 1:** Descriptive statistics of the variables and testing for differences between the groups

Variables	Group 1 (n = 68, parities < 5)	Group 2 (n = 66, parities ≥ 5)	*p*
Age, mean ± SD	47.94 ± 8.07	48.15 ± 6.42	0.552^*^
BMI, mean ± SD	28.71 ± 3.54	30.19 ± 3.82	0.440^*^
Disease duration (months), mean ± SD	5.55 ± 2.67	10.54 ± 5.22	< 0.01^*^
VAS score, mean ± SD	6.50 ± 1.61	8.42 ± 1.34	< 0.01^*^
ODI score, mean ± SD	18.41 ± 7.97	29.87 ± 6.75	< 0.01^*^
Modic change, n (%)			
None	17 (25)	4 (6.1)	** < 0.05**^**#**^
Type 1	37 (54.4)	30 (45.5)	
Type 2	14 (20.6)	32 (48.5)	
Type 3	0 (0.0)	0 (0.0)	
Schmorl’s nodes, n (%)			
None	46 (67.6)	26 (39.4)	** < 0.0**^**&**^
Yes	22 (32.4)	40 (60.6)	
Sacral slope, mean ± SD	41.01 ± 8.42	39.29 ± 7.21	0.373^*^
Pelvic tilt, mean ± SD	15.24 ± 7.31	15.05 ± 6.08	0.906^*^
Pelvic incidence, mean ± SD	62.67 ± 11.17	58.90 ± 10.30	0.156^*^
L1-S1 lordosis angle, mean ± SD	53.36 ± 12.02	55.62 ± 12.80	0.459^*^
L1-L2 lordosis angle, mean ± SD	3.86 ± 2.75	4.39 ± 2.73	0.431^*^
L2-L3 lordosis angle, mean ± SD	7.23 ± 3.54	6.95 ± 3.91	0.757^*^
L3-L4 lordosis angle, mean ± SD	10.68 ± 4.41	11.23 ± 6.17	0.675^*^
L4-L5 lordosis angle, mean ± SD	20.27 ± 7.27	18.03 ± 6.98	0.203^*^
L5-S1 lordosis angle, mean ± SD	21.80 ± 7.13	21.72 ± 7.41	0.963^*^

*ODI* Oswestry Disability Index, *VAS* visual analog scale, *BMI* body mass index

^*^Independent t test

^#^Fisher Freeman Halton Test

^&^Chi-Square test

There was no significant relationship between the distributions of patients in Group 1 and Group 2 regarding the absence of spondylolisthesis and the presence of Grade 1 and Grade 2 spondylolisthesis. A significant majority of the patients in Group 1 and Group 2 did not have spondylolisthesis (82.4% and 75.8%, respectively). In contrast, Grade 1 and Grade 2 spondylolisthesis was seen in about one-fifth of the patients in Group 1 and Group 2. Grades 3–5 were not seen in any patient (Table 2).

**Table 2:** Presence of spondylolisthesis in the groups

	Group 1	Group 2	p^*^
Not spondylolisthesis	56 (82.4%)	50 (75.8%)	0.507
Grade 1 spondylolisthesis	12 (17.6%)	14 (21.2%)	0.712
Grade 2 spondylolisthesis	0 (0.0%)	2 (3.0%)	0.306

*Chi-Square test

The descriptive statistics and the comparison of the groups' results regarding gravidity are given in Table 3. According to these results, the gravidity groups' mean age and mean BMI differences were statistically not significant (*p* = 0.074 and *p* = 0.191, respectively). The difference among the groups regarding the disease duration was statistically more significant (*p* < 0.01). The increase in number of gravidities increased with the duration of the disease. In terms of VAS and ODI scores, there were more significant differences among the groups (both *p* < 0.01), with the highest mean VAS and ODI scores being observed in the group with five or more gravidities (8.16 ± 1.49 and 28.28 ± 7.62, respectively) and the lowest scores in the single gravidity group (5.20 ± 1.92 and 11.80 ± 3.96, respectively). The number of gravidities increased with the increasing VAS and ODI scores. The relationship between gravidity and Modic changes was also statistically significant (*p* < 0.05), while the relationship between the presence of Schmorl’s nodes and gravidity was not significant (*p* = 0.075). The gravidity groups' differences in other variables were not statistically significant.

**Table 3:** Descriptive statistics and comparison of the results regarding gravidity

Variables	Gravidity = 1	Gravidity = 2–4	Gravidity ≥ 5	*p*
Age, mean ± SD	46.40 ± 3.84	46.94 ± 8.82	48.64 ± 6.93	0.074^£^
BMI, mean ± SD	27.20 ± 1.90	28.91 ± 4.45	29.89 ± 3.49	0.191^£^
Disease duration (months), mean ± SD	3.60 ± 2.64^**a**^	4.64 ± 3.14^**a**^	9.77 ± 5.01^**b**^	** < 0.01**^**£**^
VAS score, mean ± SD	5.20 ± 1.92^**a**^	6.24 ± 1.14^**a**^	8.16 ± 1.49^**b**^	** < 0.01**^**£**^
ODI score, mean ± SD	11.80 ± 3.96^**a**^	16.47 ± 6.11^**a**^	28.28 ± 7.62^**b**^	** < 0.01**^**£**^
*Modic change, n (%)*				
None	4 (40.0)	8 (23.5)	8(8.9)	< 0.05*
Type 1	6 (60.0)	20 (58.8)	40 (44.4)	
Type 2	0 (0.0)	6 (17.7)	42 (46.7)	
Type 3	0 (0.0)	0 (0.0)	0 (0.0)	
*Schmorl’s nodes, n (%)*				
None	10 (100.0)	20 (58.8)	42 (46.7)	0.075*
Yes	0 (0.0)	14 (41.2)	48 (53.3)	
Sacral slope, mean ± SD	39.66 ± 5.55	40.59 ± 9.71	40.05 ± 7.41	0.962^£^
Pelvic tilt, mean ± SD	12.94 ± 6.97	15.60 ± 7.47	15.22 ± 6.45	0.736^£^
Pelvic incidence, mean ± SD	62.90 ± 10.29	62.67 ± 9.67	59.89 ± 11.40	0.611^£^
L1-S1 lordosis angle, mean ± SD	58.08 ± 5.47	54.38 ± 12.64	54.11 ± 12.93	0.798^£^
L1-L2 lordosis angle, mean ± SD	3.50 ± 1.54	3.51 ± 2.23	4.42 ± 2.99	0.450^£^
L2-L3 lordosis angle, mean ± SD	8.00 ± 2.59	7.04 ± 3.18	7.01 ± 4.02	0.855^£^
L3-L4 lordosis angle, mean ± SD	11.92 ± 5.17	10.68 ± 4.56	10.95 ± 5.68	0.903^£^
L4-L5 lordosis angle, mean ± SD	23.98 ± 6.46	20.27 ± 6.38	18.21 ± 7.37	0.179^£^
L5-S1 lordosis angle, mean ± SD	20.90 ± 6.72	25.37 ± 6.94	20.48 ± 7.05	0.055^£^

There is no statistically significant difference between the groups indicated with the same letter in the same column (*p* > 0.05). The lettering was made according to the results of Duncan's multiple-range test. Significant p values are written in bold

*BMI* body mass index, *ODI* Oswestry Disability Index, *VAS* visual analog scale

^#^Fisher Freeman HaltonTest

^£^ANOVA the comparison of the groups with significant differences was performed using DUNCAN multiple comparison test

*Independent *t* test

## Discussion

This study investigated differences between women with grand multiparity and those with non-grand multiparity plus uniparous status experiencing lower back pain (LBP). The findings indicated a higher prevalence of Modic changes, Schmorl’s nodes, and LBP in grand multipara women. Additionally, significant variations among groups were observed in key variables such as the Visual Analog Scale, The Oswestry Disability Index, and disease duration, enhancing the overall robustness of the study. Furthermore, our conclusions affirm that parity does not influence sagittal balance parameters, including lumbar lordosis, sacral slope, pelvic incidence, and pelvic tilt.

Smith et al. has identified an association between parity, pregnancy and back pain in younger women [28]. Serdar et al. [29] found that parity is associated with degenerative disc disease and vertebral endplate changes. They also found that Schmorl's nodes were significantly associated with parity. Modic changes and degenerative disc disease were less common in grand multipara and multipara young women than in primipara women in their cross-sectional study. The authors also indicated that low parity might be associated with the development of spinal degeneration. Bailey et al. [30] have found that parity did not have an independent relationship with lumbar disc degeneration, lumbar bone mineral disease. Our study found that women with more parity, especially more than five histories of pregnancy, have a higher risk of developing degenerative disc disease and vertebral endplate changes. Modic changes and the presence of Schmorl’s nodes are among the most common degenerative changes shown in MRI in women who experienced more pregnancy history.

The mechanism behind parity as a potential risk factor for degenerative changes in spinal alignment is unknown. Pregnancy is biomechanically burdensome and creates unique loading demands on the spine that can have long-term consequences on spinal health. Pregnancy creates a period of high loading on the spine and changes in the position of the abdominal muscles [31], followed by a period of sudden loading that can disrupt the relationship between the active and passive stabilizing components of the lumbar spine, resulting in instability and pain. Recent studies on astronauts have shown that sudden spinal loading exposure changes significantly affect disc health, pain, and posture [32]. Similar sudden shifts in spinal loading may occur in postpartum women, creating a mismatch in active and passive stabilizing elements. This imbalance can lead to postural degeneration with time and if not corrected. Jeannie et al. [33] reported that parity was positively related to the deterioration of sagittal balance parameters. The authors showed associated changes in spinopelvic alignment with decreased lumbar lordosis and an increased pelvic incidence as parity increased. The effect of parity on pelvic ligament laxity may also affect postpartum sagittal alignment. It has been shown that pregnancy affects the ability to stabilize the pelvis, and this effect continues up to eight weeks postpartum [34]. Specifically, unconscious co-activation of the transverse abdominis and internal oblique muscles with the pelvic floor muscles is compromised in pregnant and postpartum women [35]. Bailey et al. [36] reported that or any of the individual sagittal balance parameters. In our study, we observed that parity did not cause any change in sagittal balance parameters.

A significant correlation was found with BMI in osteoarthritis of the facet joint. More specifically, a significant association was found at the L4-L5 level. A marginally significant relationship with BMI was found in disc narrowing and spinal stenosis. Previous studies have described the association between being overweight and an increased risk of disc degeneration [37–39]. Sebastien et al. [40] found that the relationship between being overweight and the relatively vertical inclination of the S1 end plate is preparatory for the anterior translation of L4 over L5. The posterior tilt of the pelvis suggested a compensation mechanism in patients with a high pelvic incidence and normal lumbar lordosis. As a result of the degenerative disc disease and posterior pelvic tilt, segmental lordosis was decreased at the L4-L5 and L5-S1 levels. Lordosis was increased at levels above L4-L5 as compensation for decreased caudal lordosis and the excess weight that could increase the posterior stress on facet joints. Also, sagittal-oriented and osteoarthritic facet joints did not preserve anterior shear forces and subsequent vertebral displacement. Our study found no statistically significant difference between the groups regarding BMI.

There are few studies on the MRI phenotypic features of spinal degeneration. It has been reported that multiparity increases the risk of degenerative spondylolisthesis by causing deficits in the pelvic and abdominal muscles. Each pregnancy increases the risk of developing degenerative spondylolisthesis by 22% [41]. Paul and Robert found that the incidence of degenerative spondylolisthesis was significantly higher in women who gave birth than in nulliparous women [42]. Ha et al. found a significant increase in estrogen receptors in the facet joint cartilage of patients with degenerative spondylolisthesis [43]. Another proposed hypothesis is differences in lumbar lordosis [44], sacral inclination, pelvic incidence, etc. This hypothesis relates to differences in posture between genders, including differences in posture. In the present study, no relationship between grand multiparity and spondylolisthesis could be established.

There were several limitations to our study. First, we did not evaluate the facet joints. Second, despite most of our cohort, a few of our patients had not undergone menopause yet. Moreover, the questionnaires for this study did not include information on vaginal or caesarian births. Finally, the surgical disruption of abdominal muscles during a caesarian section may have had a greater effect on postural stability; however, that could not be distinguished in this population and will require further research.

## Conclusion

In the present study, LBP was associated with parity and was observed more frequently in grand multipara women. We showed that the parameters related to low back pain increase as the number of pregnancies and births increases. Whether this situation is related to women’s hormonal status should be supported by further studies. We also concluded that parity did not change the sagittal balance parameters (lumbar lordosis, sacral slope, pelvic incidence, and pelvic tilt) and did not cause spondylolisthesis.

## Data Availability

No datasets were generated or analysed during the current study.
